# High expression of micro RNA-135A in hepatocellular carcinoma is associated with recurrence within 12 months after resection

**DOI:** 10.1186/s12885-017-3053-7

**Published:** 2017-01-18

**Authors:** Johann von Felden, Denise Heim, Kornelius Schulze, Till Krech, Florian Ewald, Björn Nashan, Ansgar W. Lohse, Henning Wege

**Affiliations:** 1 I. Department of Internal Medicine, University Medical Center Hamburg-Eppendorf, Martinistr. 52, 20246 Hamburg, Germany; 2 Institute of Pathology, University Medical Center Hamburg-Eppendorf, Martinistr. 52, 20246 Hamburg, Germany; 3 Department for Hepatobiliary and Transplant Surgery, University Medical Center Hamburg-Eppendorf, Martinistr. 52, 20246 Hamburg, Germany

**Keywords:** HCC, Liver resection, miRNA, Recurrence

## Abstract

**Background:**

Hepatocellular carcinoma has a dismal prognosis due to recurrence rates of up to 70% after curative resection. Early recurrence is driven by synchronous microscopic intrahepatic metastases. The predictive value of histological parameters is discussed controversially and adjuvant therapy is not established. The aim of this study was to identify patients at high risk for early intrahepatic recurrence by expression profiling of selected micro RNAs.

**Methods:**

In 52 patients undergoing HCC resection between 2011 and 2014, liver and tumor tissue was collected during surgery. Twelve patients with incomplete data regarding HCC recurrence, secondary liver transplantation, or perioperative death were excluded, leaving 40 patients with early recurrence <12 months (R+) or without recurrence for >24 months (R-) to compare grading, T, L, V, and R status. If tissue quality permitted, micro RNAs were measured in HCC and liver tissue.

**Results:**

Ten women and 30 men (64.0 ± 10.2 years) were analyzed. R+ occurred in 29 patients 6.2 ± 4.5 months after resection. Surveillance of R- was 26.2 ± 5.2 months. High intratumoral expression of miR-135a was associated with high risk of recurrence (HR = 4.2, *p* = 0.024, time to recurrence 8.8 ± 2.0 vs. 24.8 ± 4.4 months in patients with low miR-135a expression). As expected, T3 status was correlated with early recurrence, while other histological parameters and expression of miR-21, miR-122, and miR-125a did not.

**Conclusions:**

We show a significant association between high expression of miR-135a and early HCC recurrence. Therefore, high intratumoral miR-135a expression might serve as a novel biomarker to identify patients urgently requiring adjuvant therapy post resection.

**Electronic supplementary material:**

The online version of this article (doi:10.1186/s12885-017-3053-7) contains supplementary material, which is available to authorized users.

## Background

Hepatocellular carcinoma (HCC) is the fifth most common neoplasm in men and the third most frequent cause of cancer death worldwide [1]. HCC is staged according to the Barcelona Clinic Liver Cancer (BCLC) classification system. Very early and early stage HCC (BCLC 0 and A) are treated with liver resection (LR), ablation, or transplantation [1]. Despite these potentially curative treatment options, 50–70% of patients suffer from intrahepatic recurrence of HCC after LR or ablation, thus resulting in an overall dismal prognosis [2]. Two patterns of intrahepatic recurrence have been established, dividing the group into early (up to 2 years after LR) and late recurrence (more than 2 years after LR). In the majority of cases, early recurrence is caused by microscopic metastatic spread before LR, while late recurrence is driven by the oncogenic environment of the underlying chronic liver disease and most likely instigated by de novo transformation. Along this line, vascular invasion (macroscopic invasion by imaging or microscopic invasion in histopathology) and/or non-anatomical resection on the one hand, and multiplicity of tumor nodules and/or poor degree of differentiation on the other hand, are controversially debated as risk factors for early and late recurrence of HCC. Recently, new markers, including specific gene signatures, have been explored to stratify the risk of recurrence and to improve management of HCC, however, predictive biomarkers are still urgently needed [3, 4].

### Micro RNAs

Micro RNAs (miRNAs) are small non-coding RNAs involved in several physiological and pathological processes, such as cell growth and differentiation, inflammation, and carcinogenesis by regulating gene expression on a post-transcriptional level [5, 6]. Initially, miRNAs are transcribed as primary miRNAs, processed into precursor miRNAs, and exported to the cytoplasm to form small miRNA duplexes. The passenger miRNA is typically degraded, while the guiding miRNA strand is incorporated into the RNA-induced silencing complex (RISC) to mediate messenger RNA (mRNA) degradation or translational inhibition depending on the base pairing between miRNA and target mRNA [7, 8]. Regarding tumor formation, miRNAs may function as oncogenes or tumor suppressor genes, depending on the target mRNA and its functional role in carcinogenesis [9]. In chronic liver disease, numerous miRNAs have been investigated with regard to fibrogenesis, activity of viral hepatitis, and HCC formation [10]. Several miRNAs and their function in HCC have recently been reviewed [8, 11]. Micro RNA 21 (miR-21) belongs to the most widely overexpressed miRNAs in cancer and acts as an oncogene [12]. In HCC, miR-21 upregulation is associated with cellular proliferation and tumor growth via AKT/ERK [13]. Also, because its expression is inversely correlated with its target gene programmed cell death 4 (PDCD4), miR-21 is involved in cell migration and tumor invasion [14]. Micro RNA 122 (miR-122) is liver specific and plays a role in hepatocyte differentiation, viral hepatitis, and HCC development as a tumor suppressor. MiR-122 is linked to migration, invasion, angiogenesis, and intrahepatic metastasis via ADAM17, a transmembrane protease involved in inflammation, cellular regeneration, and cancer development [15, 16]. Micro RNA 125b (miR-125b) acts primarily as a tumor suppressor in HCC by targeting Bcl-2 and inducing cancer cell apoptosis [17]. It also inhibits cell migration and invasion by targeting LIN28B, and has recently been connected to epithelial-mesenchymal transition in HCC [18, 19]. The functional role of micro RNA 135a (miR-135a) in hepatocarcinogenesis has lately been characterized in several studies. Interestingly, Liu et al. showed that overexpression of miR-135a favours an invasive and metastatic behaviour of HCC in vitro and is associated with malignant portal vein thrombosis in vivo, most likely by directly targeting metastasis suppressor 1 (MTSS1) [20]. Two other studies showed that miR-135a also down-regulates Krüppel-like factor 4, up-regulates the expression of matrix metalloproteinase-2 and Akt, and down-regulates forkhead box O1, resulting in higher proliferation and invasiveness of HCC cells [21, 22].

### Aim

Reliable markers to predict the risk of early intrahepatic recurrence after complete surgical removal of HCC are urgently needed, especially to advance the development of adjuvant therapies, and to improve overall outcome. Based on the current paradigm that early recurrence of HCC is due to intrahepatic microscopic cancer dissemination, the aim of this study was to evaluate selected miRNAs potentially regulating invasion and metastatic spread in the resected HCC tissue.

## Methods

### Clinical specimens

**Fig. 1:**
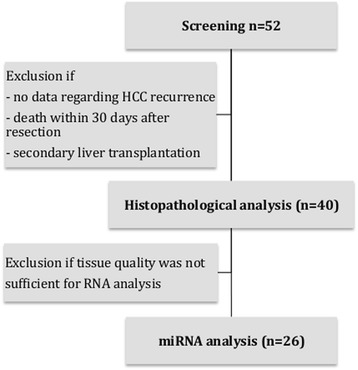
Study design. Displayed are the numbers of patients in each stage of the study and reasons for exclusion

The local ethics board approved the study (PV3578). We included 52 patients suffering from early stage HCC who underwent partial LR at the University Medical Center Hamburg-Eppendorf, Hamburg, Germany between May 2011 and May 2014. Prior to sample collection, we obtained informed consent. Patients with incomplete data regarding HCC recurrence, secondary liver transplantation, or death within 30 days after LR were excluded. Further analysis was conducted in 40 patients. Histopathology results were obtained from the Institute of Pathology, University Medical Center Hamburg-Eppendorf, Hamburg, Germany. All patients had histologically proven HCC. A flow chart of the study design with the number of patients at each stage and reasons for exclusion is displayed in Fig. 1.

### Healthy liver controls

Liver tissue from three patients who underwent LR for benign tumors (focal nodular hyperplasia or adenoma) served as healthy liver controls. Liver tissue of these patients was free from fibrosis and steatosis was <20%.

### Tissue collection, total RNA isolation, reverse transcription, and quantitative expression analysis

Following LR, HCC tissue and tissue from the surrounding non-cancerous liver were immediately transferred to Allprotect tissue reagent (Qiagen, Hilden, Germany) and stored at -80 °C until further examination. Total RNA including miRNAs was isolated in a pairwise fashion from HCC tissue and the surrounding non-cancerous liver tissue using the miRNeasy Mini Kit (Qiagen). Concentration of total RNA was measured with NanoDrop technology (Thermo Fisher Scientific, Waltham, MA, USA). Total RNA was reverse transcribed to cDNA using the miScript II RT Kit (Qiagen). Quantitative polymerase chain reaction (qPCR) was performed in a 384-well plate using the miScript SYBR Green PCR Kit, miScript Universal Primer (reverse primer), and the QuantiTect SYBR Green PCR Master Mix (all from Qiagen). The reaction mix contained 2.5 ng of sample cDNA. Primers against miR-21, miR-122, miR-125b, miR-135a were obtained from Qiagen. Detailed sequences of targeted miRNAs are displayed in Additional file 1: Table S2. As assay control, the housekeeper miRNA RNU6-6p was amplified. HuH7 cells, a human HCC cell line, were used to functionally validate the primers. Three patients with history of transarterial chemoembolization (TACE) prior to the resection had to be excluded because of substantial necrosis in the resected HCC.

### Statistical analysis

qPCR was performed in triplicates. Data are presented as median or mean ± standard deviation (STD). Regarding miRNA analysis, healthy liver controls were utilized as calibrator. The derived expression in each sample is displayed as fold change compared to the calibrator. Statistical analysis was performed with Fisher’s exact test, Cox regression, and Kaplan-Meier curves using IBM SPSS Statistics Version 22 (IBM, Armonk, NY, USA) and GraphPad Prism Version 4 (GraphPad Software, Inc., La Jolla, CA, USA). *P*-values below 0.05 were considered significant.

## Results

### Patient characteristics

Twelve of 52 patients were excluded from our analysis because of incomplete data regarding HCC recurrence, secondary liver transplantation, or death within 30 days after operation, leaving 40 patients for further investigation. This cohort contained 10 female and 30 male (75%) patients with a mean age of 64.6 ± 9.4 years. Fifteen (38%) patients had liver cirrhosis, 5 patients received TACE prior to surgical therapy, and 4 patients had previous LR. Etiologies of the underlying liver disease were viral hepatitis (n = 11), alcohol abuse [7], non-alcoholic steatohepatitis (NASH; 5), hemochromatosis [1], primary biliary cholangitis with secondary autoimmune hepatitis [1], and cryptogenic [15]. After LR, surveillance of patients was performed by imaging studies every three months for at least two years. Recurrence was diagnosed according to current imaging guidelines (RECIST) or confirmed by biopsy if imaging was not conclusive. Early recurrence occurred in 29 patients at 6.2 ± 4.6 months following LR. Mean surveillance of 11 patients without recurrence was 26.2 ± 5.4 months. Demographic characteristics of each group are listed in Table 1. Gender and age were equally distributed, while more patients in the early recurrence group had a history of prior treatment. The proportion of patients with liver cirrhosis was lower in the early recurrence group compared to patients without recurrence (31% vs. 55%, n.s.).

**Table 1:** Demographic characteristics of patients with and without early recurrence of HCC

	No recurrence (*n* = 11)	Early recurrence (*n* = 29)
Male (%)	8 (73%)	22 (76%)
Mean age at time of resection ± STD	69.2 ± 7.7 years	63.0 ± 9.6 years
Previous HCC treatment	2	7
Liver cirrhosis (%)	6 (55%)	9 (31%)
Child-Pugh A/B	5/1	7/2
Etiology of liver disease Alcohol/viral/NASH/other	3/2/1/5	4/9/4/12
Grade 1/2/3^a^	2/8/0	8/20/1
T 1/2/3^a^	6/4/0	9/8/11
V 0/1^a^	6/4	15/12
R 0/1^a^	10/0	25/3
Time to event	Mean surveillance 26.2 ± 5.4 months	RFS 6.2 ± 4.6 months

^a^In three patients complete histological analysis was not possible due to tumor necrosis because of prior TACE. Abbreviations: *RFS* recurrence free survival, *TACE* transarterial chemoembolization

### Correlation of histopathological results with early HCC recurrence

We investigated, whether grading and tumor staging parameters according to TNM classification were correlated with early HCC recurrence. Due to the small sample size, the analysis was performed nonparametric. As expected, T3 status, incorporating large tumors and multiple tumor nodules, was significantly correlated with early HCC recurrence compared to T1 and T2 tumors. All 11 patients with T3 status had early recurrence, while only 17 out of 27 patients with T1 or T2 status experienced early recurrence (*p* = 0.018). In our cohort, histological grading (G) and vascular invasion (V) were not significantly correlated with early recurrence (Table 2). Three patients had a positive resection margin (R1 status), and as expected, all of them developed early recurrence; one patient had an intrahepatic recurrence distant from the primary tumor, the other two patients showed intra- and extrahepatic spread at time of recurrence.

**Table 2:** Histological characteristics of patients with and without early recurrence of HCC

	No recurrence (*n* = 11)	Early recurrence (*n* = 29)	*p*-value (Fisher’s test)
T status^a^	T 1: 6 T 2/3: 4	T 1: 9 T 2/3: 19	0.122
	T 1/2: 10 T 3: 0	T 1/2: 17 T 3: 11	0.018
Grade^a^	G 1: 2 G 2/3: 8	G 1: 8 G 2/3: 21	0.493
	G 1/2: 10 G 3: 0	G 1/2: 28 G 3: 1	0.744
Vascular invasion^a^	V 0: 6 V 1: 4	V 0: 15 V1: 12	0.555

^a^In three patients complete histological analysis was not possible due to tumor necrosis after prior TACE. Abbreviations: *TACE* transarterial chemoembolization

### Correlation of miRNA expression with early HCC recurrence

**Fig. 2:**
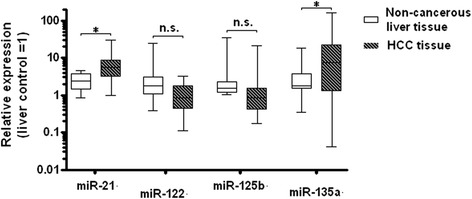
Expression levels of micro RNAs. Relative expression levels of miR-21, miR-122, miR-125b, and miR-135a in HCC tissue vs. the surrounding non-cancerous liver tissue (compared to healthy liver control, calibrated as 1). *p < 0.05

Tissue quality permitted miRNA expression analysis in 26 patients (*n* = 8 without recurrence, *n* = 18 with early recurrence). Figure 2 shows box-and-whisker-plots of the derived expression levels for miR-21, miR-122, miR-125b, and miR-135a in HCC tissue and the surrounding non-cancerous liver tissue on a log scale as fold expression compared to the calibrator (healthy liver controls). Mean expression of miR-21 and miR-135a are significantly elevated in HCC tissue vs. the surrounding non-cancerous liver tissue (6.85 ± 5.86 vs. 2.58 ± 1.22, *p* = 0.003; and 20.37 ± 34.81 vs. 3.57 ± 4.13, p = 0.04). The mean expression of miR-122 and miR-125b are decreased in HCC tissue without reaching statistical significance in this small cohort of patients (1.11 ± 0.81 vs. 3.28 ± 5.41, *p* = 0.05; and 1.99 ± 4.13 vs. 3.84 ± 7.64, *p* = 0.3).

**Fig. 3:**
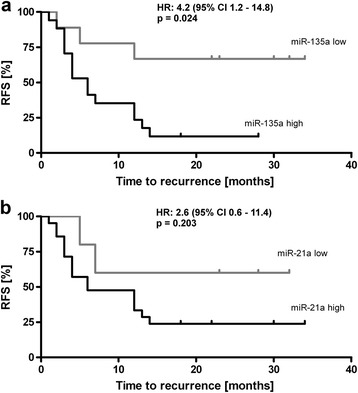
Kaplan-Meier curves. Recurrence free survival (RFS) of patients with high vs. low expression of miR-135a (**a**) and miR-21 (**b**) in HCC tissue, *n* = 26. Abbreviations: HR, hazard ratio; CI, confidence interval

The mean expression of each miRNA in the surrounding non-cancerous liver tissue was used as a threshold to divide patients into high and low expressors to allow a dichotomous and nonparametric analysis. Cox regression analysis revealed an increased hazard ratio (HR) of 4.2 with a 95% confidence interval (CI) from 1.2 to 14.8 for high expressors of miR-135a and a significance level of *p* = 0.024 (see Table 3). Figure 3a shows the corresponding Kaplan-Meier curve with a significantly lower recurrence free survival (RFS) in high expressors of miR-135a. Mean time to recurrence was 8.8 ± 2.0 months in high expressors compared to 24.8 ± 4.4 months in low miR-135a expressors. In total, 14 out of 16 patients with high expression of miR-135a had an early recurrence, resulting in a specificity of 88%. The analysis of miR-21 expression showed a similar trend with lower RFS in high expressors of miR-21, but did not reach the defined level of significance (*p* = 0.203, see Fig. 3b).

**Table 3:** Cox Regression analysis

	Hazard ratio	95% CI	*p*-value
miR-135a high	4.2	1.2–14.8	0.024
miR-21 high	2.6	0.6–11.4	0.203
miR-135a high + R-status	3.9	1.1–13.9	0.037
miR-135a high + V-status	4.2	1.2–14.7	0.025
miR-135a high + liver cirrhosis	4.2	1.2–14.8	0.024

Cox regression analysis on the influence of high expression of miR-135a and miR-21 on the recurrence of HCC, *n* = 26. Abbreviations: *CI* confidence interval

### Analysis of potential confounders and miR-135a expression

Three patients, all within the early recurrence group, were not completely resected (R1-status, see Table 2). Also, the existence of liver cirrhosis was not distributed equally between both groups (see Table 1). To investigate the influence of these potential confounders a covariate Cox regression analysis with miR-135a expression and these variables was performed showing no influence of resection status or existence of liver cirrhosis on the correlation between high miR-135a expression and early recurrence (see Table 3). As mentioned above, the widely accepted correlation of T3 status and early recurrence was also significant in our cohort. T3-status was significantly associated with high expression of miR-135a. All 10 patients with T3 showed high expression of miR-135a (*p* = 0.002) and all of these patients experienced early recurrence (100%). Within the group of patients with T1- or T2-status (*n* = 16), 5 out of 7 patients with high expression of miR-135a experienced early recurrence (71%), while early recurrence was less frequent in patients with low miR-135a expression (3 out of 9 patients, 33%). Due to the small number of patients, Cox regression analysis did not reach the defined level of significance (HR 2.9, 95% CI 0.7–12.4, *p* = 0.146). However, the Kaplan-Meier curve showed a robust trend with a mean time to recurrence of 11.6 ± 4.1 months in patients with T1 or T2 tumor status and high expression of miR-135a compared to 24.8 ± 4.4 months in patients with T1 or T2 tumor status and low expression of miR-135a (see Additional file 1: Figure S1 and Table S1).

## Discussion

The risk of recurrence is crucial for the prognosis of patients undergoing HCC resection and the most important parameter to select patients for adjuvant therapy. Therefore, we investigated putative markers to stratify the risk of early recurrence and quantified the expression of selected miRNAs in the resected HCC in comparison to the surrounding non-cancerous liver tissue.

### Patient characteristics

Age, gender, and underlying liver disease are equally contributed within our study and resemble other cohorts of patients resected for HCC [23]. The fraction of patients with liver cirrhosis is low (37%) reflecting patient selection for curative resection. Patients with large tumors and cirrhosis are not suitable for resection, while on the other hand resection is the recommended first-line treatment for patients with HCC without underlying liver cirrhosis also for large tumors [24]. In addition, a high number of patients with liver cirrhosis are not suitable candidates for resection because of portal hypertension.

### T3 and resection status are associated with early recurrence of HCC

Microvascular invasion and macroscopic multifocal tumor dissemination, especially satellite nodules near the primary tumor lesion, have been established to be predictive for HCC recurrence after resection [25, 26]. Therefore, we investigated the predictive value of histopathological markers. As expected, T3 status was significantly associated with early recurrence. However, in our small cohort of patients, microvascular invasion did not show a statistically significant association with early recurrence. However, microscopically incomplete resection (R1 status) was associated with early recurrence, underlining again the importance of complete tumor resection. Over the last years, other histopathological features have been proposed to be predictive for early recurrence. For example, a higher incidence of early recurrence or a worse outcome has been reported for tumors stained positive for cytokeratin 19, a putative stemness maker [27]. Several publications have also revealed and refined specific gene signatures predicting recurrence of HCC [4, 28]. Along this line, Villanueva et al. have proposed a composite model of clinical parameters and gene signatures to assess the risk of early recurrence in HCC [29].

### High intratumoral expression of miR-135a predicts early HCC recurrence

In our study, we investigated the expression of selected miRNAs in HCC tissue in comparison to the corresponding surrounding non-cancerous liver tissue of the same patient. We selected liver tissue from three patients without any sign of fibrosis or significant steatosis to serve as calibrator for baseline hepatic expression levels. The expression patterns of miR-21, miR-122, and miR-125b in HCC tissue and the surrounding non-cancerous liver tissue were as expected based on prior publications. A predictive value of these miRNAs regarding early recurrence of HCC was not evident in our study. In contrast, our data reveal for the first time a robust association between the risk of early HCC recurrence and high intratumoral expression of miR-135a. Noteworthy, the predictive power was independent of cirrhosis, presence of microvascular invasion, and resection status. In addition, our data demonstrate that patients with T1 and T2 tumors are more likely to develop recurrence and to have a shorter time to recurrence if miR-135a expression is increased (the data did not reach the level of statistical significance). As published recently, miR-135a is linked to vascular invasion and metastases in HCC. High miR-135a levels favour invasive and metastatic growth of HCC cells in vitro and are correlated with malignant portal vein thrombosis, possibly by directly targeting MTSS1 [20]. Other targets of miR-135a investigated in vitro are forkhead box O1, matrix metalloproteinase-2, AKT pathway, and Krüppel-like factor-4 [21, 22]. Therefore, from a functional perspective, elevated levels of miR-135a might induce early formation of microscopic HCC dissemination via several pathways, and thus, result in early HCC recurrence after curative resection. This might be especially important for patients with small tumors (T1/2) because miR-135a expression might control the risk for early intrahepatic spread.

It should be noted, that also other miRNAs have recently been described to be associated with recurrence of HCC, supporting a crucial role of these small molecules in the biology of HCC. For example, decreased expression of miR-126 in HCC has been correlated with HCC recurrence and poor survival after liver transplantation [30]. In another study, a predictive score combining the expression levels of miR-214, miR-3187 and the Milan criteria was proposed to stratify patients according to their risk for recurrence of HCC and death after liver transplantation [31].

The role of circulating miRNAs in peripheral blood for the detection of HCC or other liver diseases has also been investigated [32]. Especially serum miR-486-5p, in combination with alpha-fetoprotein levels and microvascular invasion, was found to predict risk of recurrence [33]. Following this approach, a highly sensitive digital droplet PCR of serum samples from our patients with elevated intratumoral miR-135a expression remained negative in our hands (data not shown). In contrast to other circulating miRNAs, this might be due to the very low expression of miR-135a in the tumor, resulting in only marginal quantities secreted into the peripheral blood.

### Adjuvant systemic therapy for patients undergoing resection of HCC

Adjuvant therapy with the tyrosine kinase inhibitor sorafenib was investigated in the STORM trial (Adjuvant Sorafenib for Hepatocellular Carcinoma after Resection or Ablation: A Phase 3, Randomised, Double-blind, Placebo-controlled Trial; NCT00692770). Unfortunately, the trial was negative and it is still unclear, which patients might profit from adjuvant therapy [34]. Noteworthy, patients in the STORM trial were not selected based on intratumoral biomarkers. To this regard, Hoshida et al. identified a specific expression profile in the surrounding non-cancerous liver tissue of patients undergoing resection for HCC that predicts late recurrence (>2 years). However, the study did not identify a specific expression signature within the resected HCC to predict overall recurrence or early recurrence [3, 4].

### Limitations and benefits of our study

Our study is monocentric and therefore entails only a small cohort of patients. On the other hand, we were able to derive a strong and statistically significant result for the association between high miR-135a expression and early recurrence. Our study is based on longitudinal data and the investigation of paired tissue samples from each patient (HCC tissue and the surrounding non-cancerous liver tissue).

## Conclusion

After curative intended liver resection for HCC up to 70% of patients suffer from recurrence within 2 years. Following the current paradigm, synchronous intrahepatic microscopic metastases at the time of resection, whose growth is driven by liver regeneration, are the most frequent source for early recurrence and hamper the outcome of resection. Until now, there is no established adjuvant therapy to improve this rather dismal prognosis. In conclusion, we revealed that high expression of miRNA-135a in HCC tissue correlates with a significant 4.2-fold increased risk of early recurrence and a significant reduction in time to recurrence after complete resection. Therefore, miRNA-135a might help to stratify and to identify patients who possibly benefit from adjuvant therapy after liver resection and prospective studies are needed to investigate this issue.

## Additional file

Additional file 1: Table S1. Cox Regression analysis subgroup T1 or T2. **Table S2.** Sequences of target miRNAs used in qPCR analysis. **Figure S1.** Kaplan-Meier curve subgroup analysis T1 and T2 tumors. Recurrence free survival (RFS) of patients with T1 and T2 tumor status stratified by high vs. low expression of miR-135a in HCC tissue. Cox regression *p*=0.146, *n*=16. Abbreviations: HR, hazard ratio; CI, confidence interval. (DOCX 237 kb)

## Abbreviations

ADAM17: A disintegrin and metalloprotein
AKT/ERK: Proteinkinase B/extracellular-signal regulated kinase
Bcl-2: B-cell lymphoma 2
BCLC: Barcelona clinic for liver cancer
cDNA: Coding desoxyribonucleic acid
CI: Confidence interval
HCC: Hepatocellular carcinoma
HR: Hazard ratio
HuH7: Human heptocellular carcinoma cell line 7
L: Lymphatic vessel invasion
LR: Liver resection
miR/miRNA: micro RNA
mRNA: messenger RNA
MTSS1: Metastasis suppressor 1
n.s.: Not significant
NASH: Non-alcoholic steatohepatitis
PDCD4: Programmed cell death 4
qPCR: Quantitative polymerase chain reaction
R-: No recurrence (>24 months)
R: Resection status
R+: Early recurrence (<12 months)
RECIST: Response evaluation criteria in solid tumors
RFS: Recurrence free survival
RISC: RNA-induced silencing complex 33
RNA: Ribonucleic acid
RNU6: RNA, U6 small nuclear 1
RT: Reverse transcriptase
T: Tumor size
TACE: Transarterial chemoembolization
V: Vascular invasion
