# Supplementary material for: High expression of micro RNA-135A in hepatocellular carcinoma is associated with recurrence within 12 months after resection
Source: BMC Cancer. 2017 Jan 18;17:60. doi: 10.1186/s12885-017-3053-7 (PMC5242004; doi:10.1186/s12885-017-3053-7)
Supplement: Additional file 1: Table S1. — Cox Regression analysis subgroup T1 or T2. Table S2. Sequences of target miRNAs used in qPCR analysis. Figure S1. Kaplan-Meier curve subgroup analysis T1 and T2 tumors. Recurrence free survival (RFS) of patients with T1 and T2 tumor status stratified by high vs. low expression of miR-135a in HCC tissue. Cox regression p=0.146, n=16. Abbreviations: HR, hazard ratio; CI, confidence interval. (DOCX 237 kb) [file 12885_2017_3053_MOESM1_ESM.docx]

**Additional file 1**

| **Table S1. Cox Regression analysis subgroup T1 or T2.** | | | |
| --- | --- | --- | --- |
|  | **Hazard Ratio** | **95% CI** | **p-value** |
| **miR-135a high +**  **T1/2 tumor status** | 2.9 | 0.7-12.4 | **0.146** |
| Cox regression analysis on the influence of high expression of miR-135a on the recurrence of HCC in the subgroup of patients with T1 or T2 tumor status, n=16. Abbreviations: CI, Confidence interval. | | | |

| **Table S2. Sequences of target miRNAs used in qPCR analysis.** | | |
| --- | --- | --- |
| **Target** | **Sequence** | **Catalog # (Qiagen)** |
| **hsa_miR-21_5p** | UAGCUUAUCAGACUGAUGUUGA | MS00009079 |
| **hsa_miR-122a_5p** | UGGAGUGUGACAAUGGUGUUUG | MS00003416 |
| **hsa_miR-125b_5p** | UCCCUGAGACCCUAACUUGUGA | MS00006629 |
| **hsa_miR-135a_5p** | UAUGGCUUUUUAUUCCUAUGUGA | MS00008624 |

**Figure S1. Kaplan-Meier curve subgroup analysis T1 and T2 tumors.** Recurrence free survival (RFS) of patients with T1 and T2 tumor status stratified by high vs. low expression of miR-135a in HCC tissue. Cox regression p=0.146, n=16. Abbreviations: HR, hazard ratio; CI, confidence interval.

**
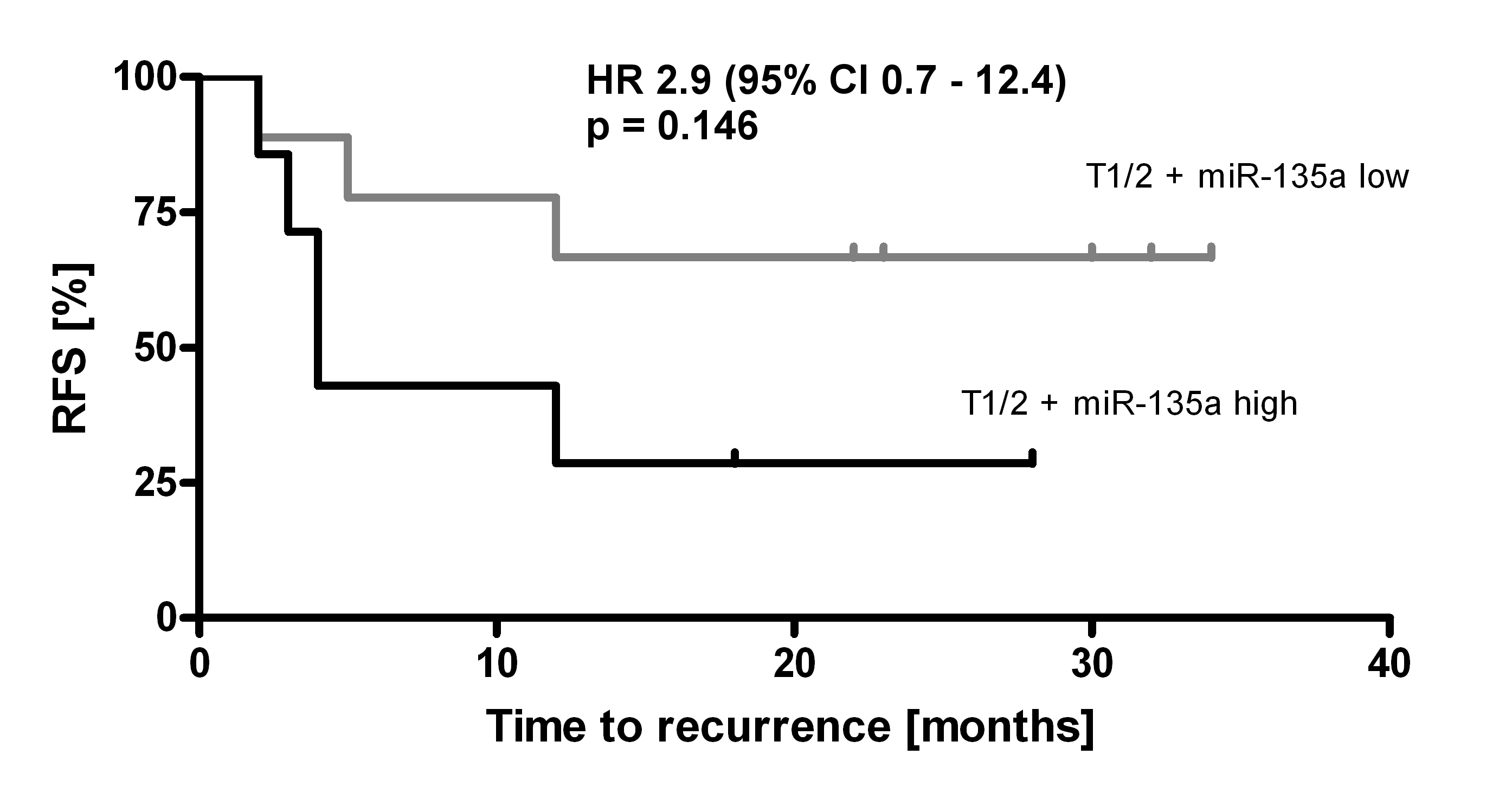
**

**Figure S1.**
